# Perspective: Gestational Tryptophan Fluctuation Altering Neuroembryogenesis and Psychosocial Development

**DOI:** 10.3390/cells11081270

**Published:** 2022-04-08

**Authors:** Xiaohong Huang, Zhendong Feng, Heng-wei Cheng

**Affiliations:** 1 Department of Pathophysiology, Institute of Neuroregeneration & Neurorehabilitation, Qingdao University, Qingdao 266071, China; zhendongfeng@hotmail.com; 2 Department of Animal Sciences, Purdue University, West Lafayette, IN 47907, USA; hwcheng@purdue.edu; 3 Livestock Behavior Research Unit, USDA-ARS, West Lafayette, IN 47907, USA

**Keywords:** tryptophan, psychosocial development, gestation, neuroendocrine, microbiota-gut-brain axis, hypothalamic-pituitary-adrenal axis

## Abstract

Tryptophan, as the sole precursor of serotonin, mainly derived from diets, is essential for neurodevelopment and immunomodulation. Gestational tryptophan fluctuation may account for the maternal-fetal transmission in determining neuroembryogenesis with long-lasting effects on psychological development. Personality disorders and social exclusion are related to psychosocial problems, leading to impaired social functioning. However, it is not clear how the fluctuation in mother-child transmission regulates the neuroendocrine development and gut microbiota composition in progeny due to that tryptophan metabolism in pregnant women is affected by multiple factors, such as diets (tryptophan-enriched or -depleted diet), emotional mental states (anxiety, depression), health status (hypertension, diabetes), and social support as well as stresses and management skills. Recently, we have developed a non-mammal model to rationalize those discrepancies without maternal effects. This perspective article outlines the possibility and verified the hypothesis in bully-victim research with this novel model: (1). Summarizes the effects of the maternal tryptophan administration on the neuroendocrine and microbial development in their offspring; (2). Highlights the inconsistency and limitations in studying the relationship between gestational tryptophan exposure and psychosocial development in humans and viviparous animals; and (3). Evidences that embryonic exposure to tryptophan and its metabolite modify bullying interactions in the chicken model. With the current pioneer researches on the biomechanisms underlying the bully-victim interaction, the perspective article provides novel insights for developing appropriate intervention strategies to prevent psychological disorders among individuals, especially those who experienced prenatal stress, by controlling dietary tryptophan and medication therapy during pregnancy.

## 1. Psychosocial Development in Adolescence

The Centers for Disease Control and Prevention (CDC, USA) reported that suicide is the second leading cause of death among children and youth between the ages of 10 and 24 during 2000–2017 [1]. The undisclosed suicide attempt is the major risk factor for completed suicides, which is correlated with the psychosocial factors in adolescents, especially among bullying victims [2]. Psychosocial problems are associated with personality disorders, social exclusion, and consequentially impaired social functioning [3,4]. The majority of the psychological disorders are thought to be caused by a person’s nature (genetic factors influencing personality characteristics), nurture (all environments affecting behavioral development), and their interactions (integrated genetic inheritance and environmental factors to physical, physiological, and behavioral development) [5]. Amongst the pregnancy-specific environments, maternal prenatal depression is associated with poor psychosocial functioning [6], and smoking during pregnancy increases the risk of attention-deficit/hyperactivity disorder (ADHD) in adolescent boys [7]. Moreover, sociological studies note that babies born to stressed mothers are easier prey for bullies [8], i.e., gestational experiences may reprogram fetal brain development and increase vulnerability to bullying. Thus, the adverse maternal-fetal transmission, e.g., maternal distress [9,10], malnutrition [11], or drug abuse [12], affecting tryptophan (Trp) metabolism, may be involved in the psychosocial disorders in offspring.

## 2. Tryptophan in Pregnancy

**Figure 1:**
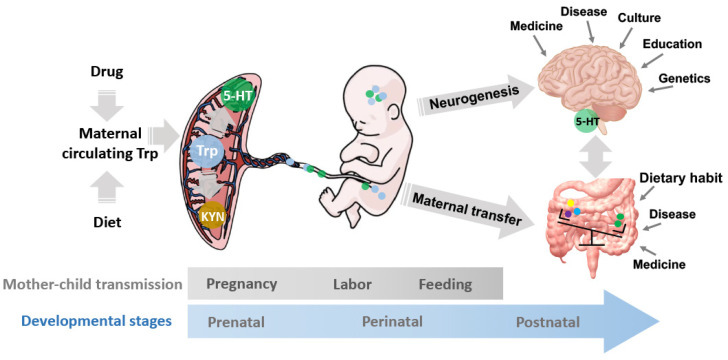
The mechanisms underlying the gestational Trp fluctuation on prenatal, perinatal, and postnatal development. The maternal circuiting Trp is affected by multiple factors, such as drug administrations and diets. The placental Trp can be synthesized to 5-HT, degraded to KYN, or transferred to the embryo. The fetus is exposed to the Trp and its metabolite, 5-HT, and KYN, which regulates the neurogenesis and neuroendocrine development as well as the gut microbiota composition and diversity in the progeny. The mother-child microbe transmissions during the 40-week pregnancy, labor, and feeding constitute the initial microbiota community in the progeny and consequentially, regulate decision making and behavioral exhibition via the MGB axis. The development and activity of the MGB axis are regulated by the maternal-fetal transfers of nutrients and microbes together with multiple environmental factors, including the disease and medicinal history, culture, eating habits, education, etc. 5-HT: serotonin; KYN: kynurenine; MGB axis: microbiota-gut-brain axis; Trp: tryptophan.

Tryptophan, an essential amino acid, is mainly derived from the diets in the small intestines and is particularly important for gestation. As the sole precursor of serotonin (5-hydroxytryptophan, 5-HT), the maternal circuiting Trp is involved in the fetal brain development directly and indirectly via the 5-HT pathway: delivered to the fetal brain and synthesized into 5-HT by tryptophan hydroxylase (TPH) 2, and synthesized into 5-HT at the placenta by TPH1, then delivered to the fetal brain (Figure 1) [13]. Serotonergic (5-HTergic) dysfunction, as one of the cellular mechanisms, is underlying the psychopathological changes including suicidality [14]. In addition, the placental Trp degrades along the cytokine-induced activated Trp catabolite (TRYCAT) pathway, producing kynurenines (KYNs) for suppression of T cell responses [15]. The altered Trp metabolism is one of the core biological signatures of prenatal maternal stress, e.g., lower plasma concentrations of Trp and KYN have been found in severe gestational anxiety and depression [10]. Antenatal stress adversely affects neuroembryogenesis via reprograming the placental Trp-KYN metabolism, which contains various neuroactive metabolites such as kynurenic acid (KYNA), 3-hydroxykynurenine (3-HK), and quinolinic acid (QUIN), laying the foundation for the neuropathology and related psychiatric disorders [9]. Moreover, urinary Trp and purine metabolites are consistently upregulated in the gestational diabetes mellitus patients [16]. Excessive increase in Trp availability compromises pregnancy by undermining T cell suppression, e.g., in pre-eclampsia [15] which is the most significant individual obstetric risk factor for schizophrenia [17]. Schizophrenia is characterized by a disturbed TRYCAT pathway in the fetal brain [18] and increased activity of systemic cortisol metabolism [19].

The excess cortisol by maternal stress crosses the placenta, affects the neuroembryogenesis, reprograms the development and activity of the neuroendocrine, and consequently induces long-lasting changes in cognitive and behavioral disorders throughout life [20]. Moreover, prenatal stress reduces the abundances of the gut Trp-metabolizing microbes in the dam and offspring, which alters the fetal exposure to Trp and its metabolites, and mediates the aberrant fetal neurogenesis, consequently reprograms microbial development in the progeny [21]. Thus, diets and or drug administrations during pregnancy may cause placental Trp fluctuation, by which it consequently alters the placental 5-HT synthesis, maternal-fetal Trp and 5-HT supply, fetal 5-HT concentrations, and gut microbiota composition as well as the endocrine and neuroendocrine developments (Figure 1). Once there, the outcomes alter the psychosocial development in offspring.

## 3. Maternal Trp Fluctuation Alters Neuroendocrine and Gut Microbiome in Offspring

To propose the perspective of gestational Trp fluctuation affecting psychosocial development in offspring, the existing evidence has been reviewed and presented in Table 1. Maternal Trp fluctuation by the Trp-enriched or -depleted diet during pregnancy alters the fetal neuroendocrine and microbiome, which may reprogram the development of the hypothalamic-pituitary-adrenal (HPA) and hypothalamic-pituitary-gonadal (HPG) axes and mediate the activity of the microbiota-gut-brain (MGB) axis in offspring, consequently reprograming the psychosocial development.

### 3.1. Maternal Trp Fluctuation Alters Psychosocial Development via Reprogramming Neuroendocrine in Offspring

Serotonin and its receptors appear early during prenatal development [37]. The fetal 5-HT, transferred from the placenta or synthesized from Trp, functions as a morphogen and tropic factor regulating brain development during embryogenesis, then as a neurotransmitter mediating the neuroendocrine functions and as a potent immune cell modulator regulating the immune system to influence multiple psychosocial events [37,38,39]. The 5-HT turnover rate in the fetal central nervous system (CNS) is at the highest stage in an animal’s entire lifespan [40]. During the prenatal and early postnatal development, 5-HT influences neural maturation and synaptogenesis [37], and disrupting 5-HT signaling leads to neurological and psychiatric disorders [41,42,43]. The alterations in the 5-HTergic system during embryogenesis have been well exemplified in pregnant women who take selective serotonin reuptake inhibitors (SSRIs), with the concerns of affecting fetal development and associated postnatal behavioral exhibitions, such as depression, anxiety, and aggressiveness [44,45].

Maternal Trp-deficient diets in pregnant mice decrease the plasma corticosterone (CORT) concentrations following restraint stress in the female adolescent offspring [36]. Thus, the maternal Trp fluctuation may alter the development and function of the HPA axis. The HPA axis is crucial in regulating the stress coping strategy confronting conflict during peer interactions [46]. The 5-HTergic neurons in the raphe nuclei regulate the HPA axis by modulating the impulses activity in the hypothalamus; ontogenetically, the 5-HTergic system regulates the synthesis of arginine vasopressin (AVP, or its non-mammalian homologue AVT, arginine vasotocin) via innervating the periventricular nucleus (PHN) and ventromedial hypothalamus (VMH) [47,48] to functionally mediate aggressiveness [49]. Equally important, maternal Trp administration in mice alters the development and function of the HPG axis during the process of pubertal maturation in offspring via regulating the productions of prolactin (PRL) and luteinizing hormone (LH) in the pituitary gland [26,28,29]. Trp-free diets from the gestation to puberty cause hypoandrogenism and hypoprolactinemia in the progeny [28,29], while maternal administration of Trp-enriched diets increases serum LH at postnatal (P) 70 d [26]. Serotonin also modulates PRL production by inducing the release of vasoactive intestinal peptide (VIP) and oxytocin (OT) from the hypothalamus [50]. A high serum PRL level is associated with psychological stress responses in humans [51]. In addition, acute psychosocial stress disrupts the LH surge in female mice [52] and central precocious puberty exerts significant effects on psychosocial development [53].

### 3.2. Maternal Trp Fluctuation Alters Psychosocial Development via Reprogramming the Gut Microbiota in Offspring

Multi-hits early-life stress alters gut microbiome, brain gene expression, and social communication, laying the foundation of the physical and mental health in adulthood [54,55]. The gut microbiota works as a virtual endocrine organ [56]. Investigating gestational Trp’s role in preventing hypertension programmed by maternal chronic kidney disease (CKD), Hsu et al. [31] found that the gestational Trp oral gavage in the CKD rats restored the abundance of microbes in offspring at P12wk, such as *Lactobacillus* and *Turicibacter*; and altered the Trp-metabolizing microbes, i.e., increased and decreased the abundance of the genus *Intestinimonas* and *Turicibacter*, respectively. The gut microbes may mediate the activity of the HPA axis via reprogramming the MGB axis. In addition, the decreased abundance of *Lactobacillus* has been characterized in the infant rhesus monkeys who experienced maternal separation and show increased stress reactivity [57]. *Lactobacillus casei strain Shirota*, a widely used probiotic strain, releases the stress-associated symptoms by suppressing the hypothalamic corticotropin-releasing hormone (CRH) and plasma CORT in the rats under water-avoidance stress [58]. Moreover, a low abundance of *Turicibacter* has been characterized in the patients with autism spectrum disorder (ASD) [59], associated with the 5-HT metabolic disorder [60]. Daily intake of *Lactobacillus helveticus CCFM1076* restores the balance of the 5-HTergic system in both the gastrointestinal tract and brain, thereby ameliorating ASD-like behaviors [61].

### 3.3. The Accompanied Physiological Alterations Are Associated with the Neuropsychological Impairment in Offspring

The physiological alterations during the offspring growth following the maternal Trp administration, including body weight (BW), blood pressure, and breath movement [22,23,24,28,29,30,31,33,34,35], may be underlying the Trp’s and 5-HT’s roles in controlling neuroembryogenesis and related 5-HTergic development in the pontomedullary brainstem [62,63]. The National Collaborative Perinatal Project (NCPP, New England) revealed a relationship between obstetrical complications and neuropsychological deficits in children aged 7 years [64]. A low birth weight is associated with neuropsychological impairments, following an index of inferred hypoxic insults [64] caused by mild to severe pre-eclampsia, maternal hyper- or hypo-tension, or maternal diabetes [15], which has been linked to the maternal Trp fluctuation [15,16,23,31]. Moreover, a survey conducted in Scania among 2000 healthy young women aged 18–34 years revealed that the women underweight have poorer psychological health compared with ones with normal BW, while the overweight women are more likely to have poor health-related behaviors and lack of internal locus of control [65]. Thus, the dissatisfaction with body image followed by improper Trp administrations may have counterbalanced mental health, causing negative influences on the psychosocial development in offspring.

## 4. Inconsistency in the Current Findings and Gaps in Knowledge

Ultimately understanding the effects of prenatal malnutrition or medicine during pregnancy on a child’s psychosocial development is a complex story. Issues are likely to cause inconsistency in the current findings, including the pregnancy stage of Trp taken, its intensity, and pregnant women’s age, eating habits, lifestyle, and healthy status. Huether et al. [61] reported that feeding a 10 g Trp-enriched standard chow powder diet daily from two weeks prior to mating through postnatal life, P4mo, resulted in a lower BW, brain 5-HT concentration, TPH2 activity, and 5-HT uptake in Wistar rats [22]. However, the administration of 30 mg/kg/day of Trp mixed with a stock chow diet for one week prior to mating increases BW at P15wk and the total 5-HT metabolite content in the medulla at P20wk in spontaneously hypertensive rats (SHRs) or deoxycorticosterone acetate (DOCA)-salt hypertensive rats [23]. Moreover, the considerations about maternal seasonality and season of conception are advocated when studying the causality between maternal Trp fluctuation and fetal neurodevelopment. Because the differences in the plasma Trp concentrations have been detected across maternal seasonality and season of conception [66]. The longitudinal studies of the Trp impacts also are needed, because the early life adversity can be buffered and masked by the favorable experience at a later age, like family support, culture, and education [67]. Taken together, a number of challenges in human subject and experimental non-human mammalian researches, including the inevitable background noise during the embryogenesis and multiple stimulations during the postnatal development, exist in unveiling the association between the maternal Trp metabolism and the psychosocial development in their progeny (Figure 1).

The National Center for Educational Statistics (NCES, Washington, DC, USA) in 2015 revealed that 21% of the students aged 12–18 years are being bullied on school premises [68]. The studies about the underlying mechanisms of bullying mainly focus on genetics and environments, while the contribution of the maternal-fetal transmission is not well clear. The 40-week pregnancy, labor (natural labor vs. cesarean section), feeding (breast-feeding vs. formula-feeding), and the fluctuation in the individual pregnancy-specific environments may affect the neurogenesis, neuroendocrine development, and gut microbiota composition and diversity in progeny, which consequently alters the development and activity of the MGB, HPA, and HPG axes, affecting the sociometric status in a peer relationship. For instance, in aberrant fetal neurodevelopment, the reduced abundances of the Trp-metabolizing microbes have been associated with the stress-disrupted placental Trp [21]. However, the mother-to-child transmission of the dietary Trp and 5-HT synthesized in the placenta as well as microbes during pregnancy, labor delivery, and breastfeeding is irresistible in viviparous animals [69], which causes inevitable background noise in studying the influences of gestational Trp fluctuation on the physiological and behavioral developments in offspring. To date, no specific animal model has been established for investigating the underlying mechanisms of the effects of mother-to-child transmission on bullying.

## 5. Evidences in a Chicken Model

### 5.1. Chicken Model

**Figure 2:**
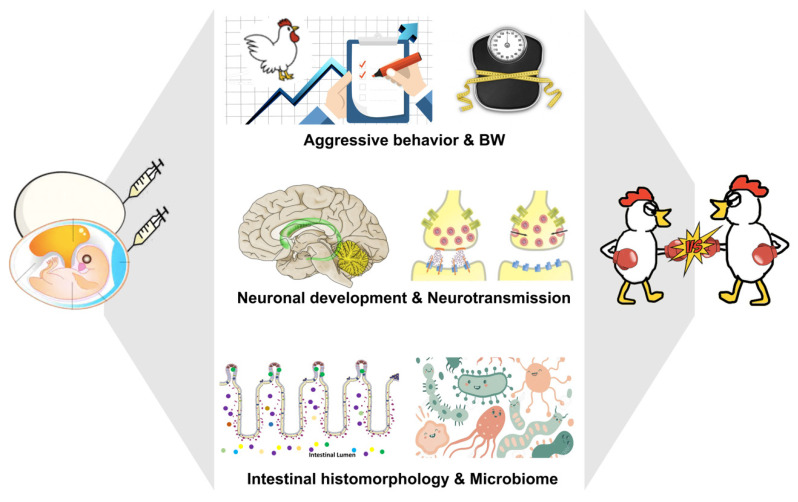
The photograph represents the gestational tryptophan fluctuation in the chicken model for bullying research. Embryonic Trp or 5-HT exposure regulates neuronal development, neurotransmission, intestinal histomorphology, and microbiota composition, consequently altering the postnatal behavioral and physiological exhibitions, which determines an individual’s psychosocial development during the lifespan. 5-HT: serotonin; BW: body weight; Trp: tryptophan.

With the significant, fundamental similarities between the human and chicken genomes [70,71], avian species have been central in investigating the subsequent cognitive, social, and neural development influenced by the experiences occurring during the ontogenic stages due to the directly controllable embryogenesis in the oviparous animals. Chicken have been used as an animal model in various clinical and psychopharmacological studies [72,73] as well as for assessing the effects of genetics, environments, and genetic-environmental interactions on psychopathological disorders [74,75]. Noteworthy, chickens are currently an elective model system in investigating the ontogenic origins of behaviors [70,74,75], like ASD [76,77]. Social behavioral deficits have been reported in the chicken model, including impaired early social predispositions [76,78], low aggregation and belongingness, and weak vocalization, when exposed to sodium valproate (VPA) during E14–E21 [77]. A laying hen produces more than 300 eggs a year with a potential for producing numerous embryos with similar genetic backgrounds and robust microbial communities at once [79] but independence of maternal influences on the neuroembryogenesis. In doing so, the chicken model has been employed in investigating the effects of embryonic exposure of Trp and its metabolite, 5-HT, on aggression and bullying in our lab (Figure 2) [74,80,81]. Within flocks, chickens have a social hierarchy referred to as a pecking order: peckers within a flock always start feather pecking at others, as a reason to avoid adding young birds, small birds, or strange birds to the flock. Hence, peckers within a flock could be a comparison to bullies within a social group.

### 5.2. Embryonic Trp Exposure Yields Bullying Victim

In ovo Trp administration (500 μg/egg) yields bullying victims in White Leghorn birds, a commercial strain [75], reducing the BW and aggressiveness in the male offspring before and during adolescence [80], resulted from the reprogrammed MGB axis. The gut histomorphology has been altered by the Trp exposure, i.e., the crypt depth is increased while the villus/crypt ratio is decreased [80]. The abundant genus, *Olsenella*, in the Trp group [80] has been identified as the core microbe in the crypt of the human colon [81]. The correlations between the gut morphological alteration and high abundance of *Olsenella* are consistent with the findings in patients with depression [82] and ASD [83]. Moreover, the abundance of *Olsenella* is increased in the lean people in the Japanese population [84], which is positively correlated with the Yale Food Addiction Scale (YFAS) used for identifying signs of addictive-like eating behavior, hedonic food intake [85]. Thus, the alteration in the gut bacterial community may be involved in determining the bodily force and regulating the neuropsychological activity facing bullying.

Peer victimization exhibits more stress with altered HPA axis activity during the trier social stress test (TSST) [86]. The HPA axis is developmentally cross-linked with the gut microbiota [87], which hints at the gut microbes’ role in neurogenesis and related behavioral development in offspring. The chicken HPA axis is functionally similar in stress response to that of mammals [88,89,90,91]. The increased catecholamine concentrations in the chicken hypothalamus, including dopamine, epinephrine, and norepinephrine, post embryonic Trp exposure may be associated with the alterations in the gut microbiome and MGB axis function, laying the foundation of their mental status confronting conflictual peer interactions [80]. Taking as an example skatole, one of the gut Trp metabolites produced by *Olsenella* [92], shows a positive association with the anatomical connectivity of the amygdala [85], by which it is involved in reward learning and addiction [93]. The amygdala is constantly scanning our environments for initiating the HPA to regulate emotional and mental reactions in responding to internal and external stimulations [94,95].

### 5.3. Embryonic Serotonin Exposure Reduces Aggressiveness in Bullies

Identical twins with ASD differ significantly in the severity of social traits [96], which is probably due to the unequal blood supply from the placenta during embryogenesis. The placenta is the primary source of 5-HT for fetal development. The promoter of 5-HT transporter (5-HTT) contains two common alleles, which are associated with the dysregulation of 5-HTergic neurotransmission: the short (S) allele has a lower transcriptional efficiency compared to the long (L) allele [97]. Bullied children with the SS genotype are at greater risk for developing emotional problems at age 12 than those children with the SL or LL genotype [97]. In humans and various animals, the 5-HTergic system regulates aggression via innervating the midbrain and hypothalamic dopaminergic neurons [98,99,100]. The midbrain dopamine storage capacity is negatively correlated with aggression [101]; individuals who have a low dopamine transmission capacity show more impulsive aggression in response to provocation [101]. The midbrain and hypothalamic 5-HTergic innervations have been evidenced in the chicken brain as well as the dopaminergic neuronal distribution and neurotransmission during mid-late embryogenesis [48]. Moreover, in ovo 5-HT injection (10 μg/egg) reduces aggressive behaviors at a cost of increased fearfulness during adolescence and before sex maturation in the White Leghorn birds [102]. The Dekalb XL birds, a highly aggressive strain [75,103], the inborn bullies, are picked to explore the effect of prenatal 5-HT exposure (10 and 20 μg/egg) on the behavioral development and underlying mechanisms [104]. Both dosages of 5-HT exposure reduce aggression at P7wk, and the 5-HT exposure effects can be achieved via the different paths by modifying the embryonic 5-HTergic and dopaminergic systems and altering fetal 5-HTergic influence on the thalamocortical circuit and HPA axis. Briefly, the 10 µg 5-HT exposure reduces the 5-HT turnover rate, increases 5-HT 1a receptor expression, and facilitates the ventral tegmental area (VTA) neuronal development, suggesting an increased 5-HT availability but not 5-HT pool potentially facilitates the midbrain neuronal development. The 20 µg 5-HT exposure enhances the 5-HTergic regulation to the hypothalamus and VMH, indicated by the increased both the 5-HTergic and dopaminergic neurotransmission [104]. Hyper-excitability of the neurons in the ventrolateral part of the VMH exaggerates aggression under certain pathological conditions, like ASD [105]. These findings shed light on the mechanisms underlying hypo- and hyper-serotonemia as the potential risk factors of ASD [106].

## 6. Conclusions

Tryptophan is required for protein synthesis for fetal development and participates in programming the neural circuits and development of the neuroendocrine system; on the other hand, excess Trp is responded to maternal inflammation to initiate signaling cascades, such as KYN metabolic pathway, to stimulate embryonic brain development. Through the mother-child transmission, maternal circuiting Trp, as one of the major gestational Trp resources, is critical for the development of MGB, HPA, and HPG axes in offspring with long-lasting impacts on physical, physiological, and behavioral outcomes. The abnormal Trp metabolism during embryogenesis has been recognized as one of the reasons in patients with emotional problems and mental diseases. Maintaining an appropriate Trp level by controlling dietary Trp and rigorous medication therapy during pregnancy has been advocated as a biotherapeutic targeting strategy for preventing psychosocial disorders, emotional problems, and mental diseases in adolescence, like bullying.

## Figures and Tables

**Table 1:** Non-comprehensive review of the effects of maternal Trp administration on the physiological and behavioral exhibitions in offspring.

Species	Treated Time	Control	Treatment	Exhibitions in Offspring	Refs
Wistar rats	14 days prior to mating–P4mo *	Standard chow powder (3.5 g Trp/kg)	10 g Trp mixed with the diet (13.5 g Trp/kg)	Decreased BW of the male offspring at P4mo; Decreased 5-HT concentration, TPH2 activity, and 5-HT uptake in the frontal cortex and brain stem.	[22]
SHRs or DOCA-salt hypertensive rats	7 continued days prior to mating	Stock chow diet	30 mg Trp/kg/day mixed with the diet	Increased BW and blood pressure during P5wk–P15wk; Increased brain weight at P20wk; Increased total 5-HT metabolite content (5-HT plus 5-HIAA) in the medulla at P20wk.	[23]
Humans	210 min	Continued fasting	1 g Trp orally	Increased incidence of fetal breathing movements; Unchanged breathing rates and breath interval variability.	[24]
SD rats	E17	Saline vehicle	200 mg Trp/kg oral gavage	Increased Trp, 5-HT, and 5-HIAA concentrations in the fetal brain at E17 and E18.	[25]
SD rats	E15–E21	Saline vehicle	200 mg Trp/kg oral gavage	Increased serum PRL at P40d and P70d; Increased serum LH at P70d; Increased forebrain 5-HT and 5-HIAA at P70d.	[26]
Wistar rats	E19 and E21	0.1 N-HCl vehicle	250 mg Trp/kg i.p.	Increased intracerebral concentrations of Trp at E19; Decreased valine, methionine, leucine, tyrosine, phenylalanine, and histidine at E19; Increased phosphoserine, threonine, serine, glutamic acid, and Trp at E21; Decreased methionine, leucine, and histidine at E21; Increased protein synthesis activity indicated by [^3^H] Leucine incorporation at E19 and E21.	[27]
SD rats	E14.5–late puberty	Control chow (0.22% Trp)	Trp free diet (0.00% Trp)	Dwarfism pups; Decreased serum GH concentration in male and female offspring; Severe hypoprolactinemia; Normal right-timed onset of puberty in both male and female rats.	[28,29]
Wistar rats	E5–E21	Regular chow diet	Trp-free diet (0.2% Trp)	Unchanged Brain weights in newborn pups; Decreased BW in newborn pups; Reduced numbers of 5-HTergic neurons at the dorsal raphe, especially at the medial and caudal sections of dorsal raphe, which contains the majority of 5-HTergic neurons; Unchanged brain 5-HT concentration.	[30]
SD rats	E1–E21	Control	200 mg Trp/kg oral gavage	Increased kidney weight-to-BW ratio at P12wk; Increase blood pressure in male offspring at P4wk, P6wk, P8wk, P10wk, and P12wk; Decreased plasma level of L-citrulline, a precursor of l-arginine and SDMA, an indirect inhibitor of NO synthase; Increased gene expressions in the AHR pathway.	[31]
CKD SD rats	E1–early postnatal life *	Control	200 mg Trp/kg oral gavage	Decreased systolic blood pressure, mean arterial pressure, and creatinine at P12wk; Decreased plasma level of L-citrulline and SDMA; Altered the abundance of the Trp-metabolizing microbes, i.e., increased the abundance of the genus *Intestinimonas* and decreased the abundance of *Turicibacter*.	[31]
SD rats	E1–late puberty	Control rat chow (0.22% Trp)	Trp free diet (0.00% Trp)	Pronounced dwarfism pups; Decreased serum GH concentration in males and females; Marked hypoandrogenism and severe hypoprolactinemia in males; Hypoprolactinemia in females; Right-timed pubertal maturation in both sexes.	[28,29]
SD rats	E1–P12wk *	Control rat chow (0.22 g Trp/100 g of pellets)	high-Trp diet (1 g Trp/100 g of pellets)	Increased blood 5-HT, i.e., hyperserotonemia during P1wk-P12wk; Decreased blood GH; Decreased TPH1 activity in gastrointestinal tracts tissue; Decreased IGF-I expression in hepatic and muscle tissue.	[32]
SD rats	E1–weaning	500 mg Trp/100 g diet	75 mg Trp/ 100 g diet	Decreased average BW at weaning; Unaffected opacities at P22d.	[33]
SD rats	E1–P25d *	TD.99366 control diet (1.8 g Trp/kg)	TD.08125 Trp-deficient diet (1 g Trp/kg)	Normal BW at P5d but reduced BW at P15d and P25d; Decreased body temperatures at P15d and P25d; Unaffected Oxygen consumption (V_O2_); Altered breathing pattern and slower heart rates at 15 d; Decreased ventilation (V_E_) and V_E_-to-V_O2_ ratios in both air and 7% CO_2_ at P25d; Increased ventilatory response to CO_2_ at P5d in male offspring and reduced at P15d and P25d in male and female offspring; Reduced medullary 5-HT concentration, while similar 5-HT neuronal number.	[34]
Pigs	Third trimester of gestation–delivery	2×Trp diet (0.26% Trp fed in the morning and afternoon)	High-low Trp diet (0.39% Trp fed in the morning and 0.13% Trp fed in the afternoon)	Decreased birth healthy pig rate and birth weight of piglet per pen with similar total birth weight per pen; Decreased serum phosphoserine, taurine, cysteine, proline in newborns and increased liver n-6:n-3 PUFA ratio; Altered gene expressions, including the genes related to cytotoxic effector regulation, NADH oxidation, ROS metabolism, and tissue development.	[35]
Outbred CD-1 mice	Lactation (P0d–P8d)	Standard laboratory diet (0.14% Trp)	Trp-deficient diet (0.00% Trp)	Unchanged time spent in open sectors in the 0-maze test in adolescent daughters (P189d–P193d); Unchanged time spent in floating in the forced-swim test in adolescent daughters; Unchanged time spent in the novel compartment in the novelty-seeking test in adolescent daughters; Unchanged achieved breakpoint in the progressive ratio operant procedure in adolescent daughters; Decreased plasma CORT concentrations and similar BDNF concentrations following restraint stress in adolescent daughters.	[36]

* The pups are fed the same diet as mothers throughout postnatal life. 5-HIAA: 5-hydroxyindoleacetic acid; 5-HT: serotonin; AHR: aryl hydrocarbon receptor; BDNF: brain-derived neurotrophic factor; BW: body weight; d: day; CKD: chronic kidney disease; CORT: corticosterone; DOCA: deoxycorticosterone acetate; E: embryonic day; GH: growth hormone; IGF-1: insulin-like growth factor-I; i.p.: intraperitoneal; LH: luteinizing hormone; mo: month; NADH: nicotinamide adenine dinucleotide; P: postnatal; PRL: prolactin; PUFA: polyunsaturated fat; SD: Sprague-Dawley; SDMA: symmetric dimethylarginine; SHRs: spontaneously hypertensive rats; TPH: tryptophan hydroxylase; Trp: tryptophan; ROS: reactive oxygen species; wk: week.
